# Clustered Incidence of Leukocytoclastic Vasculitis and Purpura Fulminans: A Case Series of a Rare Dermatological Manifestation of Rickettsial Disease

**DOI:** 10.7759/cureus.27187

**Published:** 2022-07-23

**Authors:** Christopher J Pinto, Shadab B Maldar, Sumitha Subramaniam, Naina Fathima, Rajesh Nayyar, Rina J Patel

**Affiliations:** 1 Department of Infectious Diseases, Karnataka Institute of Medical Sciences, Hubballi, IND; 2 Department of Pediatrics, Karnataka Institute of Medical Sciences, Hubballi, IND; 3 Department of Pathology and Laboratory Medicine, Karnataka Institute of Medical Sciences, Hubballi, IND; 4 Department of Medical Research, Karnataka Institute of Medical Sciences, Hubballi, IND; 5 Department of Emergency Medicine, Southern Illinois University School of Medicine, Carbondale, USA; 6 Department of Family Medicine, China Medical University, Shenyang, CHN

**Keywords:** skin lesions, rocky mountain spotted fever, travel history, rickettsial diseases, acute infectious purpura fulminans

## Abstract

Rickettsiae are a group of eukaryotic obligatory intracellular parasites with ticks and mites as vectors. *Rickettsia conorii *is the Indian counterpart of Rocky Mountain spotted fever causing the endemic variant - Indian tick typhus. This disease can cause severe illness in adults and children and can be missed despite the availability of serological tests. Initial screening for rickettsial diseases (RD) may include blood workup and a non-specific agglutination test, Weil-Felix (WF). In WF, agglutination against Proteus antigens is analyzed and can show false-negative results within the first week of presentation. Delayed immune reaction in patients with RD in the first week could also be responsible for negative specific IgM serology. The challenge for physicians is to differentiate between the two common diagnoses for fever with rash - viral exanthematous fever and rickettsial fever. By its endothelial cell tropism, RD rarely can lead to purpura fulminans, which is characterized by widespread progressive dermal vascular necrosis and hemorrhage. This case series demonstrates dermatologic presentations of rickettsial fever in three individuals from the same neighborhood within the same week. Based on serologic IgM levels, the patients were treated with doxycycline and made a full recovery. This case series aimed to highlight the need for awareness regarding the variable presentations of rickettsial fever including leukocytoclastic vasculitis and purpura fulminans.

## Introduction

Rickettsial diseases (RD) are difficult to diagnose as they present with variable symptoms across time. Symptoms may range from a fever with rash to multi-organ dysfunction and death. They are vector-borne diseases, with the vectors being ticks, louse, and mites [1]. Most patients complain of high-grade fever and a rash within one to three days following an incubation period of five to 10 days [1]. Young adults and children are more severely affected. In many cases, patients do not recall being bitten by the vector [2]. The incubation period may be generally shorter in patients who develop severe symptoms (five days or less from the time of vector bite) [2]. The counterpart of Rocky Mountain spotted fever in the Indian subregion is caused predominantly by *Orientia tsutsugamushi* and *Rickettsia conorii* which have a predominant tropism towards endothelial cells and could be responsible for small vessel vasculitis [1,3]. Clinical presentations could range from a benign fever to multiorgan failure, leading to death [1-5].

Purpura fulminans (PF) is an emergency condition characterized by tissue necrosis and disseminated intravascular coagulation. Infectious agents such as *Streptococcus pneumoniae* group A and other beta-hemolytic streptococci, *Staphylococcus aureus*, *Neisseria meningitidis*, *Candida albicans,* and Rickettsiae can cause PF [6]. PF could also be an outcome of inherited protein C or S deficiency. Biopsy and histopathology may show necrosis with neutrophilic infiltrate surrounding dermal vasculature [1,6]. Difficulty in identifying the etiologic cause and delayed treatment with doxycycline could have fatal outcomes [5]. In literature, few cases of purpura fulminans have been reported following rickettsial infections.

## Case presentation

Case 1

**Figure 1:**
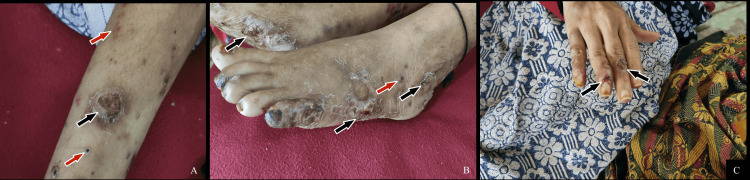
Physical findings in case 1 The images show (A-C) multiple necrotic skin lesions (marked in black) surrounded by petechiae (marked in red).

A 31-year-old Indian female from Gadag, Karnataka, was referred to our emergency department with complaints of high-grade fever, muscle pain and evolving skin rash over the past three days. Physical findings showed a febrile female with a temperature (of 39°C) with multiple adherent skin lesions on her leg and her hands that bled easily on touch (Figures 1A-1C).

**Figure 2:**
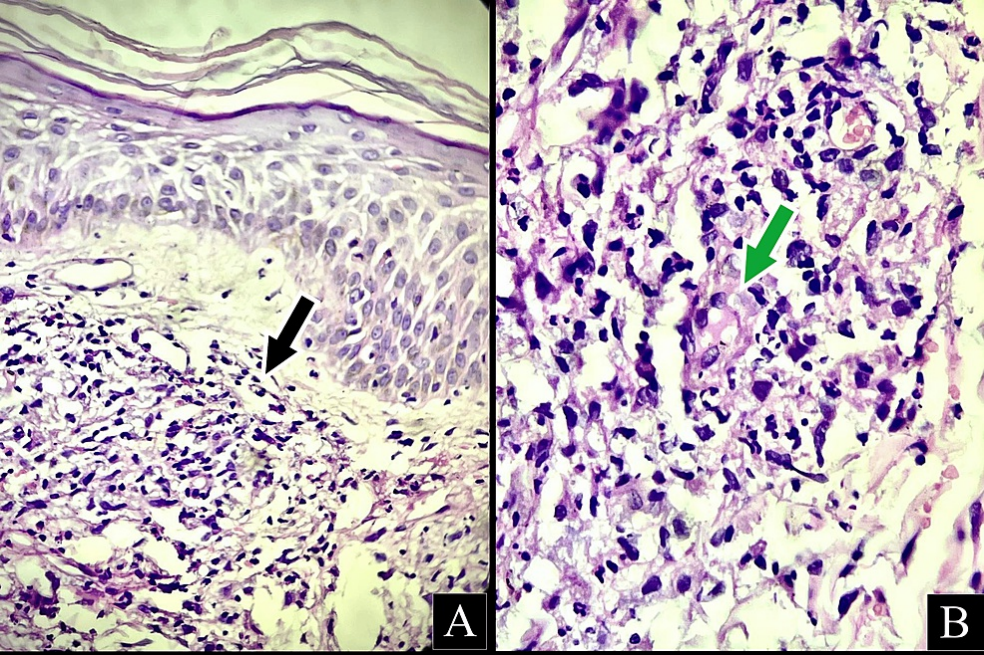
Skin biopsy findings in case 1 The images show (A) dense nuclear infiltrates along with dermal edema, epithelioid granuloma marked in black (H&E); (B) epithelioid histiocytes infiltrating a vessel (marked in green) (H&E).

Skin biopsies of the involved sites showed features typical of leukocytoclastic vasculitis (Figures 2A, 2B). Laboratory investigations were significant for low hemoglobin (11.0g/dl), leukopenia (3100 cells/dl), thrombocytopenia (150,000 cells/dl) with prolonged clotting times (activated partial thromboplastin clotting time {APTT}: 40 seconds and prothrombin time {PT}: 20 seconds). Renal function tests were within normal limits. Thin and thick smears were negative for the malarial parasite. Workups for dengue fever, leptospirosis, and viral exanthematous fevers were negative.

Serologic testing showed a Positive Weil-Felix test with further testing showing IgM-specific positivity (Table 1). Based on the positive serology, the patient was started on oral doxycycline. The patient's temperature was tracked and showed resolution of lethargy and fever on inpatient day five.

**Table 1:** Laboratory panels for cases 1, 2, and 3 Hb: hemoglobin; APTT: activated partial thromboplastin time; PT: prothrombin time; SGOT: serum glutamic oxaloacetic transaminase; SGPT: serum glutamic pyruvic transaminase; DIC: disseminated intravascular coagulation

Cases	Blood counts	Reference range (N)	Inference	Serology on day 1	Serology on day 3
Case 1	Hb: 11.0 g/dl	12-14g/dl	Mild anemia	Weil Felix - positive	-
	WBC: 3100 cells/dl	-	Leukopenia		
	Platelet: 150,000/dl	-	Low platelet count		
	APTT: 40 seconds	30-40 seconds	Slightly prolonged	Rickettsial fever-specific IgM - positive	-
	PT-INR: 20 seconds	11-13.4 seconds	Slightly prolonged		
	SGOT: 90.23U/l and SGPT: 78.03U/l	0-41U/l and 0-55U/l	Mildly elevated liver enzyme values		
Case 2	Hb: 6.8 g/dl	12-14g/dl	Severe anemia	Weil Felix - negative	-
	WBC: 3650 cells/dl	-	Leukopenia		
	Platelet: 43,000/dl	-	Findings suggestive of DIC		
	APTT: 80 seconds	30-40 seconds		Rickettsial fever-specific IgM - positive	-
	PT-INR: 45 seconds	11-13.4 seconds			
	SGOT: 230U/l and SGPT: 175U/l	0-41U/l and 0-55U/l	Moderately elevated liver enzyme values		
Case 3	Hb: 7.3 g/dl	12-14g/dl	Severe anemia	Weil Felix - negative	-
	WBC: 3800 cells/dl	-	Leukopenia		
	Platelet: 48,000/dl	-	Findings suggestive of DIC		
	APTT: 60 seconds	30-40 seconds		Rickettsial fever-specific IgM - negative	Rickettsial fever-specific IgM - positive
	PT-INR: 30 seconds	11-13.4 seconds			
	SGOT: 35U/l and SGPT: 22U/l	0-41U/l and 0-55U/l	Normal liver enzyme values		

Case 2

A six-year-old male child from Gadag, Karnataka, was referred to our pediatric emergency department with complaints of high-grade fever (39.7°C), lethargy, muscle pain, and vomiting with an expanding rash. Alert surveillance system including epidemiology indicated that this was the second incidence of febrile exanthematous illness within the same neighborhood within the same week. Cases 1-3 lived in the same neighborhood within 300 m of each another.

**Figure 3:**
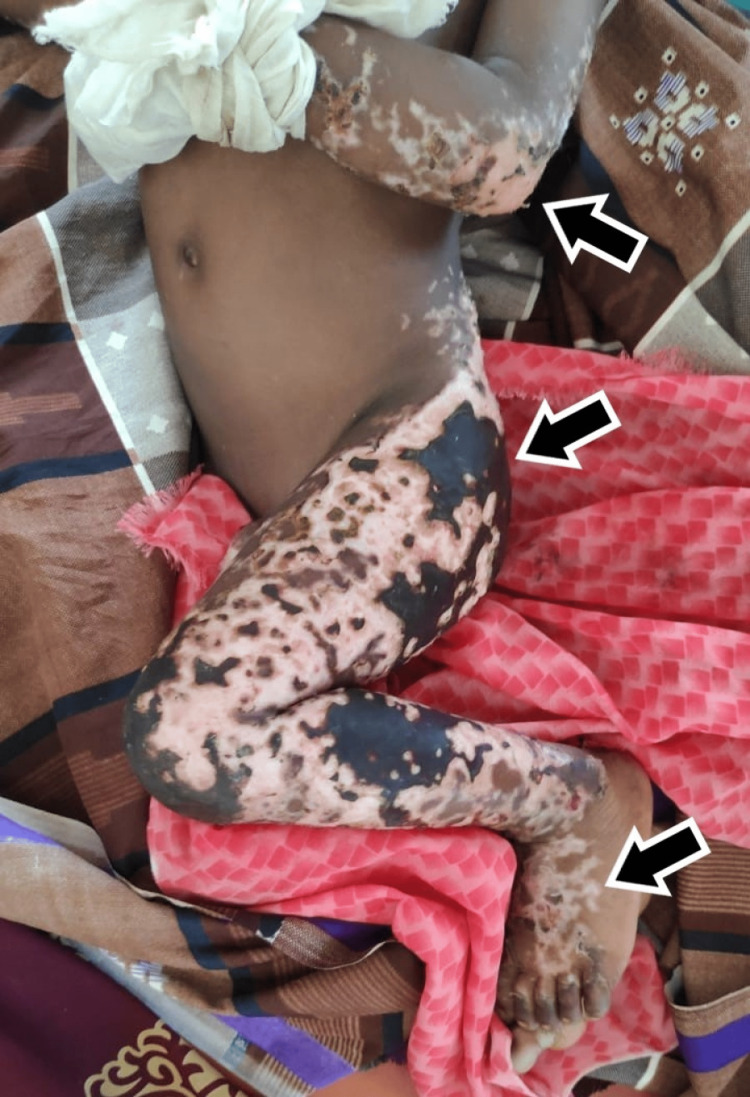
Physical findings in case 2 The image shows ecchymoses and necrotic involvement of elbows, buttocks, legs, and feet.

**Figure 4:**
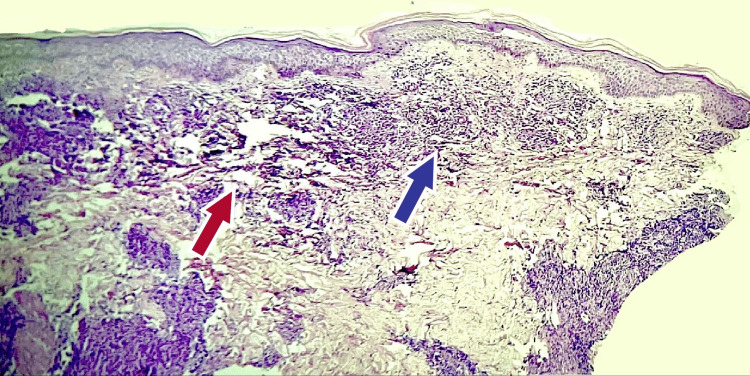
Skin biopsy findings in case 2 The image shows neutrophilic nests (marked with blue) with sub-dermal micro-abscesses (marked in red) (H&E).

Physical findings showed widespread skin lesions sensitive to touch involving the lower limbs, buttocks, and lower half of the back, elbows, and fingertips as seen in Figure 3. Skin biopsy of the involved sites showed nests of neutrophilic infiltration with subdermal microabscesses suggestive of purpura fulminans as seen in Figure 4. Laboratory investigations were significant for severe anemia (6.8 g/dl), leukopenia (3650 cells/dl), thrombocytopenia (43,000 cells/dl), with clotting times suggestive of disseminated Intravascular coagulation (APTT: 80 seconds and PT: 45 seconds) with deranged liver function. Renal function tests were within normal limits. Thin and thick smears were negative for malarial parasites. Workups for dengue fever, leptospirosis, and viral exanthematous fevers were negative.

Serologic testing showed a negative Weil-Felix reaction; however, serologic testing for Ricketssial specific IgM antibodies was positive as seen in Table 1. Based on the positive serology for IgM antibodies for rickettsial antigens, the patient was started on oral doxycycline. The patient could not tolerate oral medications and hence was switched to IV doxycycline 2 mg/kg twice daily. This was continued till the patient could tolerate oral medications. Prolonged clotting times and low platelet counts were suggestive of disseminated intravascular coagulation (DIC), which was treated with low molecular weight heparin and fresh frozen plasma for the first two days [7]. Wound care was through regular applications of topical antibiotics and prophylactic amoxicillin-clavulanic acid. The patient’s temperature was tracked and showed resolution of lethargy and fever on inpatient day 10 with scaling on skin.

Case 3

A three-year-old male child from Gadag, Karnataka, was referred to our pediatric emergency department with complaints of high-grade fever (38.8°C), lethargy, muscle pain, and vomiting with an expanding rash. Alert surveillance system including epidemiology indicated that this was the third incidence of febrile exanthematous illness within the same neighborhood within the week.

**Figure 5:**
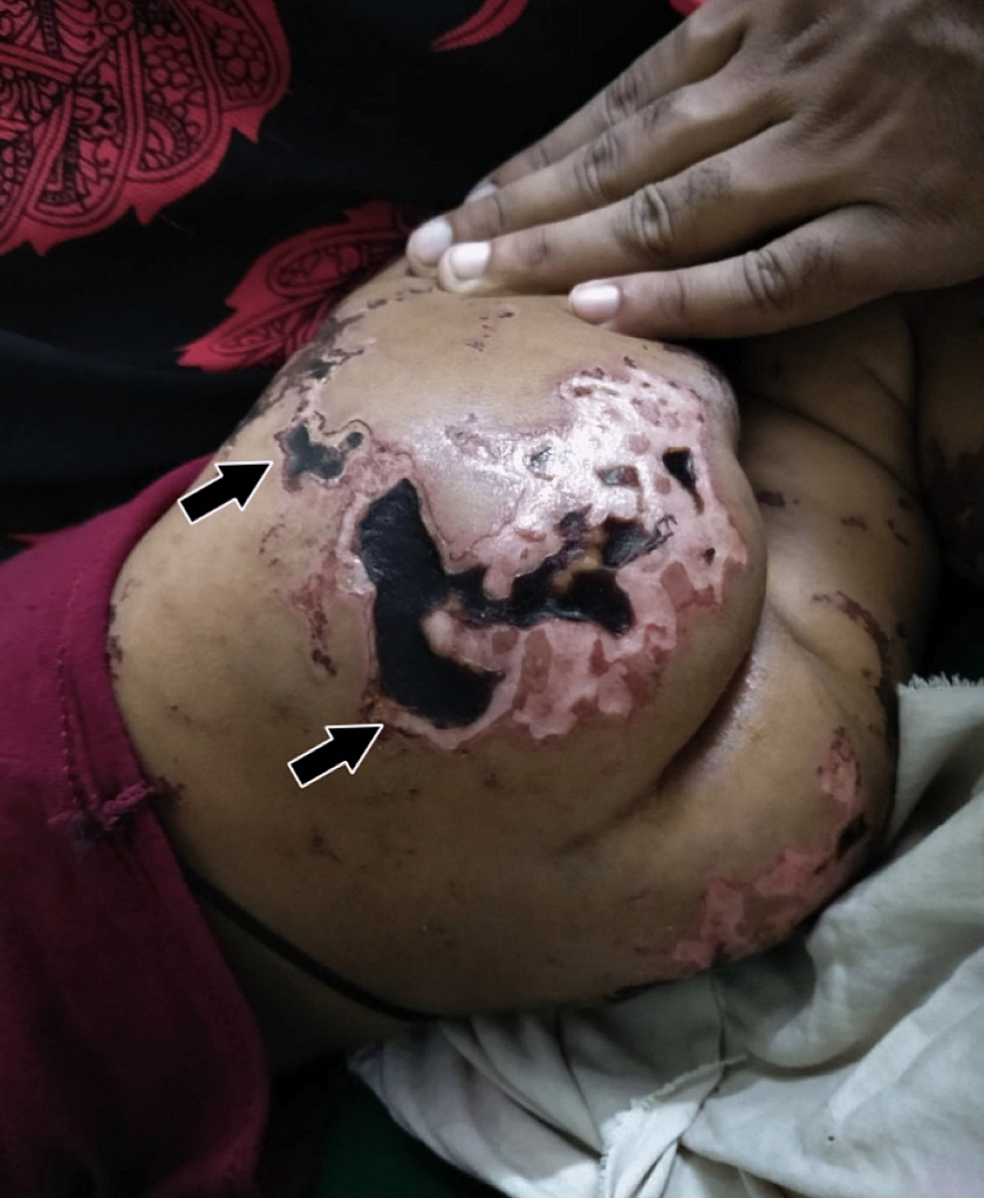
Physical findings in case 3 The image shows superficial necrosis involving the buttocks.

**Figure 6:**
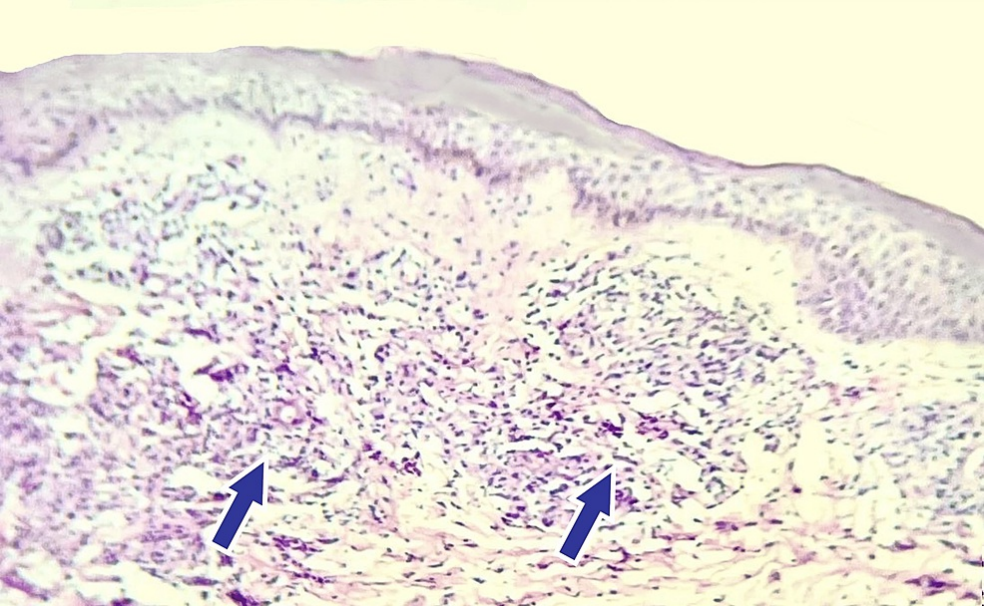
Skin biopsy findings in case 3 The image shows neutrophilic nests (marked with blue) suggestive of purpura fulminans (H&E).

Physical findings showed widespread skin lesions sensitive to touch involving the lower limbs, buttocks, and lower half of the back seen in Figure 5. No involvement was seen in the upper extremities. Skin biopsy of the affected areas showed nests of leukocytic infiltration as seen in Figure 6 suggestive of purpura fulminans.

Based on the similar presentations in cases 1 and 2, the patient was started on oral doxycycline. A false-negative result of specific IgM serology on day one was suspected to be based on early presentation. A clustered incidence in cases 1 and 2 helped in early diagnosis, as day three serology showed positive blood serology for IgM antibodies. Prolonged clotting times and low platelet counts were suggestive of DIC, which was treated with low molecular weight heparin and fresh frozen plasma for the first two days [7]. Calamine lotion was provided to soothe the inflamed areas. The lesions in this patient showed significant improvement on day five of inpatient care and started scaling. The patient’s temperature was tracked and showed resolution of fever on inpatient day eight. A summary of the presenting complaints and skin biopsy findings of cases 1, 2, and 3 are present in Table 2.

**Table 2:** Symptoms and physical findings results of the skin biopsy for cases 1, 2, and 3

Cases	Symptoms	Physical findings	Skin biopsy findings
Case 1	High-grade fever (39°C)	Multiple adherent skin lesions that bleed easily when probed. Multiple punctate hemorrhages seen surrounding the larger lesions. Lesions were present on the hand and on the lower limbs	Dermal edema with cellular infiltrate around proliferating capillaries indicative of leukocytoclastic vasculitis
	Muscle pain		
	Vomiting		
	Expanding rash		
Case 2	High-grade fever (39.7°C)	Skin lesions were sensitive to touch. Dense wide patches of inflamed and necrotic skin tissue widely distributed encompassing both the lower limbs, buttocks, lower half of the back, elbows, and fingertips	Nests of leukocytic infiltration with subdermal micro-abscesses suggestive of purpura fulminans
	Lethargy		
	Muscle pain		
	Vomiting		
	Expanding rash		
Case 3	High-grade fever (38.8°C)	Skin lesions were sensitive to touch. Dense wide patches of inflamed and necrotic skin tissue widely distributed encompassing the lower limbs, buttocks, and lower half of the back	Nests of leukocytic infiltration suggestive of purpura fulminans
	Muscle pain		
	Vomiting		
	Expanding rash		

## Discussion

Rickettsial diseases (RD) are difficult to diagnose due to variable presentations with different periods of onset [1]. Usual clinical features of RD include myalgia with vomiting, lethargy, fever, and an evolving maculopapular rash involving the palms and soles. Leukocytoclastic vasculitis seen in case 1 showed a benign course with a maculopapular rash. Patients may show prolonged fever, renal failure, and progression of depleting platelets to disseminated intravascular coagulation. Evolution of the disease in uncommon presentations shows severe abdominal pain, acute respiratory distress syndrome, myocarditis, and neurological deficits isolated or in conjunction with meningoencephalitis [4,8,9].

Purpura fulminans (PF) is an emergency condition characterized by widespread tissue necrosis and hemorrhage. It could be of an inherited deficiency of protein S, protein C, and antithrombin III [5]. Acquired or idiopathic origin could also cause PF. The more documented cause is infections of *S. pneumoniae* group A and other beta-hemolytic streptococci, *S. aureus*, *N. meningitidis,* and *C. albicans* [5-7]. Rickettsial diseases have been documented to be a rare etiologic cause of PF [5-7,9]. Irrespective of the cause, the presence of well-defined symmetrical necrotic ecchymotic lesions with coagulation abnormalities (DIC) is typical of PF [7]. The dermatological manifestations could be considered as an outcome of an exaggerated immune response towards the endothelial cell trophic rickettsial organisms. Hence, variable presentations in a 31-year-old, six-year-old, and three-year-old were seen. The dog tick *Rhipicephalus sanguineus* (vector) was suspected as the implicated cause of the clustered incidence within the same neighborhood [10,11].

In cases 2 and 3, coagulation studies showed prolonged values with anemia (consumptive DIC), hence requiring heparinization and fresh frozen plasma [7]. Biopsy and histopathology may show necrosis with neutrophilic infiltrate surrounding dermal vasculature [12]. Workup of ecchymotic skin rashes with high-grade fever, myalgia, and lethargy should include testing for RD and coagulation studies [1,2]. Serological tests used in confirmation are indirect immunofluorescence IgM-specific antibodies towards rickettsial antigens, Western blot, and polymerase chain reactions [5]. Cross-reactivity of immunoglobulins is noted between *Rickettsia rickettsii* and other Rickettsiae (*R. conorii*) and hence a positive result is a titer greater than 1:64 [13]. Weil-Felix (WF) test can be used preliminary screener as it is an effective, economical tool. But WF herein failed to show any reactivity in case 2, wherein IgM antibody titers were significant and hence were unreliable here in the early phase of the disease [14,15]. The titer of IgM required for positive serology (1:64) can also take up to 10 days from the onset of illness and could be responsible for false negatives [1,2]. Moreover so, the clustered incidence in cases 1 and 2 helped guide the diagnosis of rickettsial disease-induced purpura fulminans in case 3. Rickettsial fevers are easily treatable but untreated cases can have a mortality of up to 35% [4]. Delayed treatment with doxycycline in the presence of negative WF and negative serology has shown to cause multiorgan dysfunction and death [4,5,16].

Other differential diagnoses with preceding symptoms of myalgia could push towards a viral exanthematous infectious cause for the pyrexia of unknown origin delaying further treatment. Rashes with an infectious cause would have the differentials of roseola, dengue, malaria, leptospirosis, *N. meningitidis,* and drug-induced thrombocytopenic purpura. But many of these exanthematous fevers avoid the palms and soles; on the contrary, RD can involve the palms and soles [1,2].

Mainstay treatment of RD includes the use of doxycycline (tetracycline class) - orally or through IV by binding to the bacterial ribosomal 30s subunit. Previously known statements regarding the use of tetracyclines in children are no longer substantiated due to widespread studies showing no association between its use and bone deformities/teeth discoloration [1]. Hence usage is safe for a short period of 10-14 days and does not pose any threat. Chloramphenicol could also be used as an alternative but is associated with multiple side effects that require constant monitoring [1,2]. Use of azithromycin in the dosage of 10 mg/kg/day is permitted in cases wherein doxycycline and chloramphenicol have adverse drug reactions [17].

## Conclusions

Rickettsial diseases are difficult to diagnose due to variable presentations with different periods of onset. Workup of ecchymotic skin rashes with high-grade fever, myalgia, and lethargy should include testing for Rickettsial disease and coagulation studies. Delayed treatment in the presence of negative Weil-Felix and negative serology could cause multiorgan dysfunction and death. Hence, early identification through clustered incidences may help with identification of the disease. Early treatment of Rickettsia-induced purpura fulminans and leukocytoclastic vasculitis with doxycycline improves patient outcomes and recovery.
